# Current Treatment Patterns and Outcomes of Sex Cord Stromal Tumor Patients in Japan

**DOI:** 10.1111/iju.70399

**Published:** 2026-03-10

**Authors:** Takashige Abe, Kosuke Miyai, Toyonori Tsuzuki, Sachiyo Murai, Katsuyoshi Hashine, Shinichi Yamashita, Takashi Kawahara, Naotaka Nishiyama, Hiroshi Kitamura, Hiroshi Kikuchi, Kazutaka Saito, Hatsuki Hibi, Haruka Miyata, Ryuji Matsumoto, Takahiro Osawa, Hiroyuki Nishiyama, Nobuo Shinohara

**Affiliations:** ^1^ Department of Urology Hokkaido University Graduate School of Medicine Sapporo Japan; ^2^ Department of Basic Pathology National Defense Medical College Saitama Japan; ^3^ Department of Surgical Pathology Aichi Medical University Hospital Nagakute Japan; ^4^ Department of Urology National Hospital Organization Shikoku Cancer Center Ehime Japan; ^5^ Department of Urology Tohoku University Graduate School of Medicine Sendai Japan; ^6^ Department of Urology, Faculty of Medicine University of Tsukuba Tsukuba Japan; ^7^ Department of Urology University of Toyama Toyama Japan; ^8^ Department of Urology Dokkyo Medical University Saitama Medical Center Saitama Japan; ^9^ Department of Male Reproduction Minato Medical Coop‐Kyoritsu General Hospital Nagoya Japan

**Keywords:** pathological risk factor, prognosis, sex cord stromal tumor, survival, treatment

## Abstract

**Objectives:**

Sex cord‐stromal tumors (SCSTs) constitute approximately 5% of testicular malignancies. This investigation aimed to elucidate contemporary therapeutic approaches and clinical outcomes for patients with testicular SCSTs in Japan.

**Methods:**

Participating institutions included Japan Clinical Oncology Group facilities, affiliated centers, and institutions with published cases. Clinical data and histopathological findings were compiled. Central pathological review involved representative tissue sections when available. Kaplan–Meier methodology assessed recurrence‐free survival (RFS) and overall survival (OS). Stage I patients were stratified by cumulative pathological risk factors (tumor diameter ≥ 5 cm, necrosis, moderate to severe nuclear atypia, lymphovascular invasion, infiltrative growth, and ≥ 3 mitoses per 10 high‐power fields).

**Results:**

Among 116 patients from 66 institutions, 112 met inclusion criteria. Histological distribution included Leydig cell (*n* = 54), Sertoli cell (*n* = 36), and other variants (*n* = 22). Twelve patients presented with metastatic disease; ten received systemic chemotherapy, with 20.8% five‐year OS. Among 91 stage I patients with follow‐up data, eleven developed recurrence. Patients with > 2 risk factors demonstrated significantly inferior RFS compared with those showing ≤ 1 factor (26.4 versus 98.6% at 5 years, *p* < 0.0001). Surgical intervention predominated in the recurrent cases (81.8%, 9/11), while chemotherapy was administered to 45.5% (5/11), and selected patients achieved prolonged disease control through repeated surgical resection without systemic therapy. OS rates were 90.9% and 50.5% at 5 and 10 years.

**Conclusions:**

Patients with synchronous metastatic presentation demonstrated poor survival. Stage I patients with > 2 pathological risk factors showed reduced RFS, with surgical management potentially efficacious for recurrent disease.

## 1 Introduction

Sex cord‐stromal testis tumors (SCSTs) represent approximately 5% of all testicular neoplasms [1], with roughly 10% exhibiting malignant behavior [2]. Among these, Leydig cell tumors predominate, followed by Sertoli cell tumors. Given the rarity of this disease, clinicians frequently extrapolate disease management established for testicular germ cell tumors. Although recent reports have involved retrospective cohort analyses utilizing comprehensive national databases [3] and systematic reviews of published cases [4, 5, 6], optimal therapeutic strategies remain undefined, particularly regarding the implementation of retroperitoneal lymph node dissection (RPLND) and appropriate chemotherapeutic protocols for metastatic disease. To elucidate the current treatment landscape and optimize management strategies for both localized and metastatic SCSTs, the collection of individual patient data, including pathological review, is important. This investigation had dual objectives: primarily, to delineate current treatment patterns and outcomes among Japanese SCST patients (clinical part); secondarily, to establish a digital histopathological repository to improve diagnostic accuracy and advance pathological expertise (pathological part). In this report, we present the clinical outcomes.

## 2 Patients and Methods

The study methodology was devised by Japanese Urological Oncology Group (JUOG TC2101). After receiving institutional review board approval (Hokkaido University Hospital Life and Medical Research Ethics Review Committee, No.: 022–0073), we extended invitations to Japan Clinical Oncology Group (JCOG)‐affiliated institutions and their associated medical centers, alongside facilities with published case studies in Pubmed or Ichushi‐Web, the Japanese biomedical literature repository (https://search.jamas.or.jp/search). Patients' baseline data including age, presenting symptom, serum tumor marker measurements if available, histopathological findings, treatments, disease recurrence, and final outcomes were collected. When feasible, two representative pathology sections (10 unstained slides per section) were voluntarily submitted for central assessment and integration into the digital pathology database. The histological specimens underwent evaluation by two uropathologists (KM and TT). For cases with unusual histologic features or uncommon tumor types, a panel of immunohistochemistry was assessed, including inhibin, steroid factor‐1, melan A, calretinin, β‐catenin, CD30, synaptophysin, chromogranin, and/or SALL4. The antibodies used and their technical specifications are summarized in Table S1. Their central pathological assessment superseded original diagnostic reports in cases of discrepancy.

Pathological indicators of aggressive disease were systematically categorized utilizing both original pathological documentation and contemporary histological review where available. These prognostic indicators comprised tumor dimensions exceeding 5 cm, presence of tumor necrosis, moderate to severe nuclear atypia, lymphovascular invasion, infiltrative growth, and greater than 3 mitotic figures per 10 high‐power fields. Originally, Kim et al. compared pathological features of benign (*n* = 14) and malignant (*n* = 5) Leydig cell tumors and defined six features [2]. Then, these 6 histopathological criteria were utilized for other SCST subtypes [7, 8]. Overall survival (OS) was defined as the interval between the date of surgery for testis and that of death. Recurrence‐free survival (RFS) was calculated from the date of surgery for testis to disease recurrence or death from any cause. The univariate cox model was utilized to assess the impact of each abovementioned adverse pathological characteristic and age (continuous) on RFS. RFS was also stratified according to the cumulative number of aforementioned pathological risk factors, with comparison among groups by the log‐rank test. Statistical analyses were conducted utilizing JMP Student edition 18.2.0 (SAS Institute, Cary, NC, USA). Significance was established at *p* < 0.05.

## 3 Results

**FIGURE 1:**
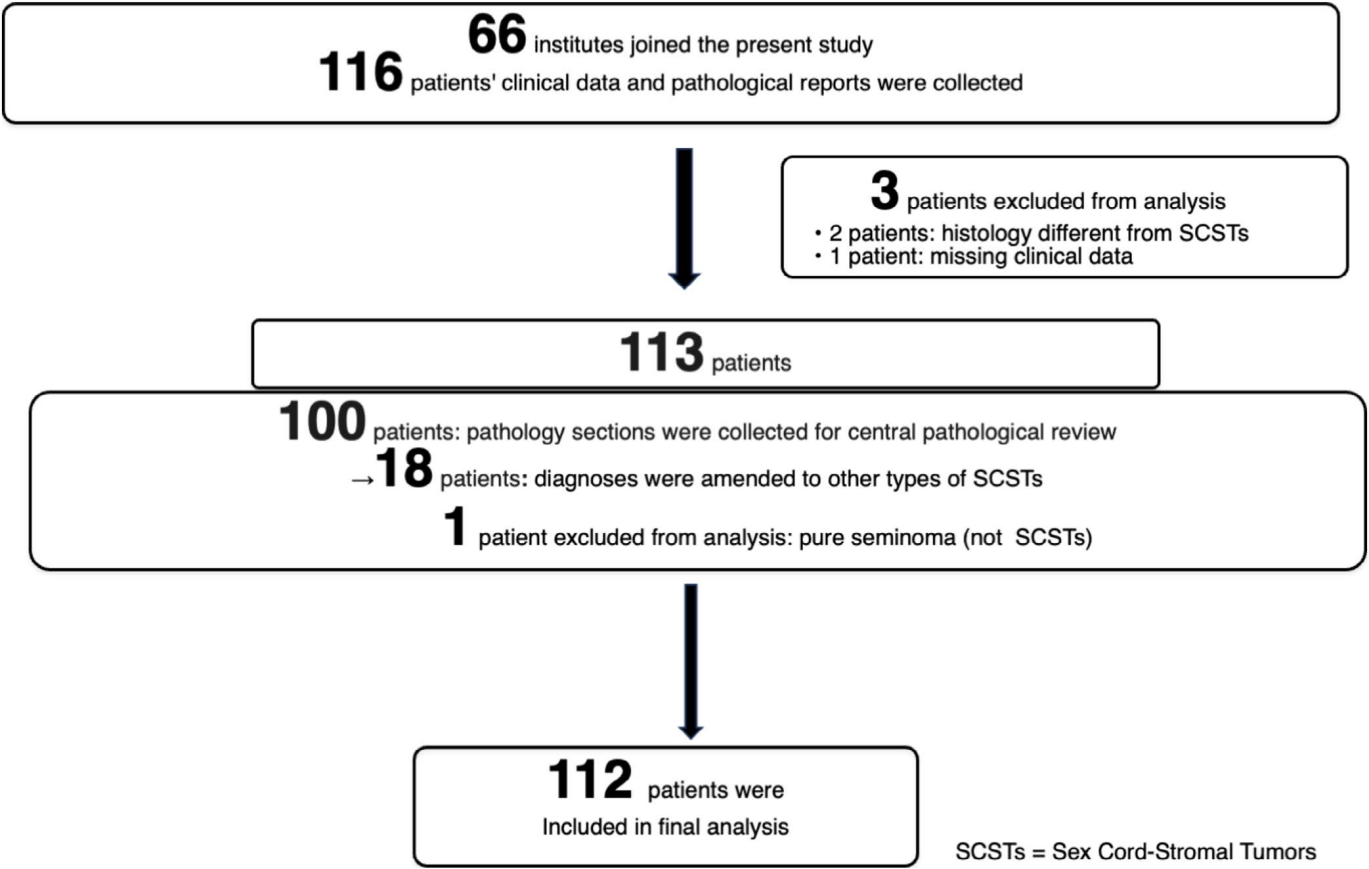
Patients' selection flow.

Figure 1 summarizes patients' selection flow. Sixty‐six institutes participated in the present study. Overall, 116 patients' clinical data and pathological reports were collected. Of those, two patients were excluded due to the histology different from SCSTs, based on the original pathological report, and one due to most clinical data being missing. Pathology sections were collected for central assessment in 100 patients, and pathological diagnoses were amended to other types of SCSTs in 18 patients. After excluding 1 patient whose histology did not indicate SCSTs based on central pathological review (pure seminoma), 112 patients were included in the current analysis.

### 3.1 Demographics, and Clinical Symptoms

**FIGURE 2:**
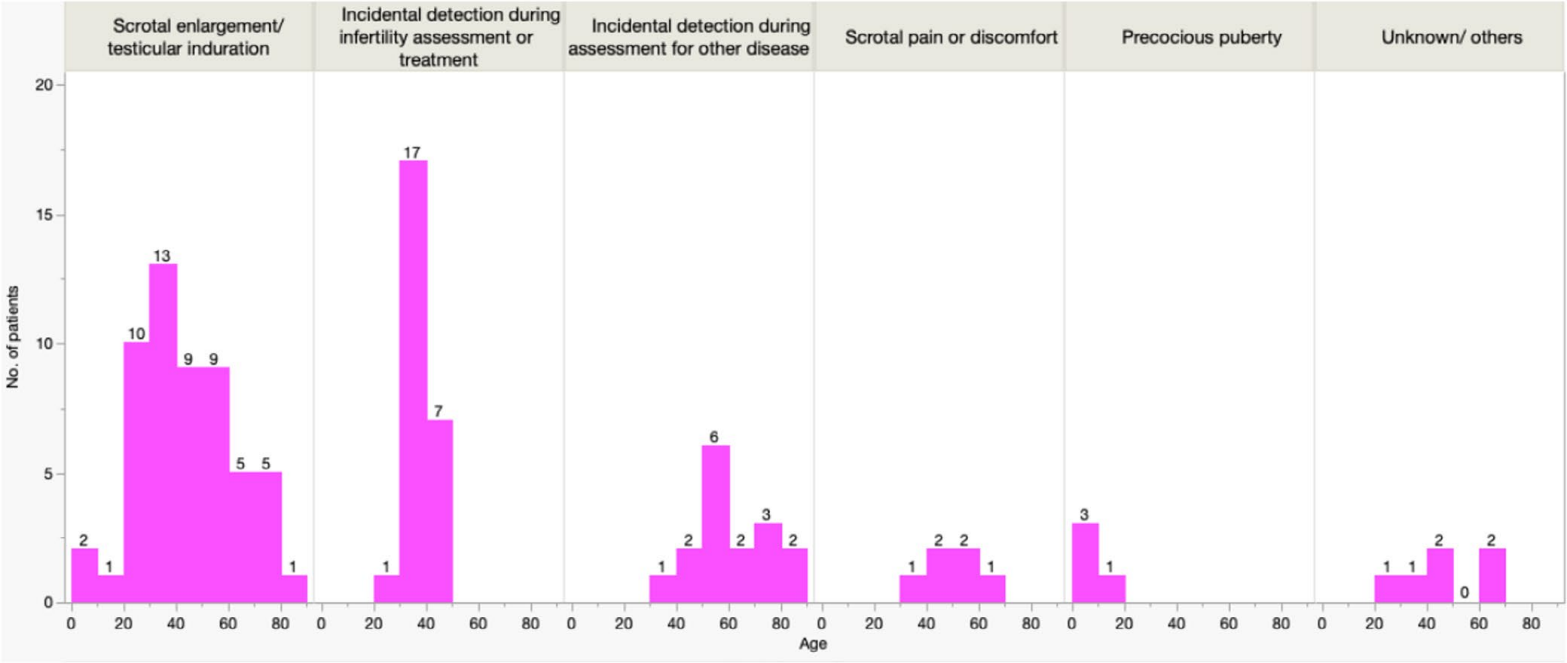
Summary of age groups at diagnosis divided by clinical symptoms.

Table 1 summarizes the patients' characteristics. The median age at diagnosis was 42 years (range: 0–85). In terms of age groups at diagnosis divided by clinical symptoms (Figure 2), the majority presented with scrotal enlargement/testicular induration (55/112, 49.1%), showing a mountain‐shaped distribution with peaks in the 20s and 30s, followed by incidental detection during infertility assessment or treatment (25/112, 22.3%), showing a steep peak in the 30s. Precocious puberty was also reported in pediatric cases (4/112, 3.5%). Twelve patients had metastatic disease at diagnosis [retroperitoneal lymph nodes (RPLNs) = 5, lung = 3, lung+bone = 2, RPLNs+distant LNs = 1, brain+lung+RPLNs = 1].

**TABLE 1:** Patients' characteristics.

	Total, *n* = 112	Patients without metastasis at initial diagnosis, *n* = 100	Patients with metastasis at initial diagnosis, *n* = 12
Age (years), median (range)	42 (0–85)	41 (0–85)	48.5 (24–80)
Laterality:Right/Left/Bilateral	47/61/4	39/58/3	8/3/1
Clinical signs and symptoms			
Scrotal enlargement/testicular induration	55	48	7
Incidental detection during infertility assessment or treatment	25	25	0
Incidental detection during assessment for other disease	16	13	3
Scrotal pain or discomfort	6	5	1
Precocious puberty	4	4	0
Unknown/others	6	5	1

### 3.2 Local Treatment and Pathological Findings

Table 2 summarizes the local treatment and pathological findings. Most patients underwent radical orchiectomy with/without testicular sperm extraction (95/112, 84.8%). Testis‐sparing surgery was performed in 16 cases (16/112, 14.3%). On pathological assessment, 54 patients had Leydig cell tumors, 36 had Sertoli cell tumors, 5 had large cell calcifying Sertoli cell tumors, 4 had Sertoli‐Leydig cell tumors, 3 had inflammatory and nested sex cord tumors, and 10 had other histologies.

**TABLE 2:** Summary of local treatments and pathological findings.

	Total, *n* = 112	Patients without metastasis at initial diagnosis, *n* = 100	Patients with metastasis at initial diagnosis, *n* = 12
Surgery for testis			
Radical orchiectomy	89	77	12
Radical orchectomy+microdissection TESE	6	6	0
Partial orchiectomy/tumor enucleation	8	8	0
Microdissection TESE+partial orchiectomy/tumor enucleation	8	8	0
Resection of intra‐abdominal testis	1	1	0
Pathology			
Leydig cell tumor	54	52	2
Sertoli cell tumor	36	33	3
Large‐cell calcifying Sertoli cell tumor	5	4	1
Sertoli‐Leydig cell tumor	4	4	0
Inflammatory and nested testicular sex cord tumor	3	1	2
Mixed or unclassified sex cord‐stromal tumor/others	10	6	4
Tumor size 5 cm or more Yes/No/Unknown	24/82/6	17/79/4	7/3/2
Lymphovascular invasion Yes/No/Unknown	18/91/3	10 87/3	8/4/0
≥ 3 mitotic features per 10 high‐power fields Yes/No/Unknown	19/91/2	12/86/2	7/5/0
Nuclear atypia Yes/No/Unknown	16/92/4	9/88/3	7/4/1
Necrosis Yes/No/Unknown	19/90/3	9/88/3	10/2/0
Infiltrating margins Yes/No/Unknown	13/95/4	7/89/4	6/6/0
No. high‐risk features abovementioned, median (range)	0 (0–6)	0 (0–5)	4 (0–6)

Abbreviation: TESE, Testicular sperm extraction.

### 3.3 Treatments and Outcomes of Metastatic Disease at Initial Presentation

**FIGURE 3:**
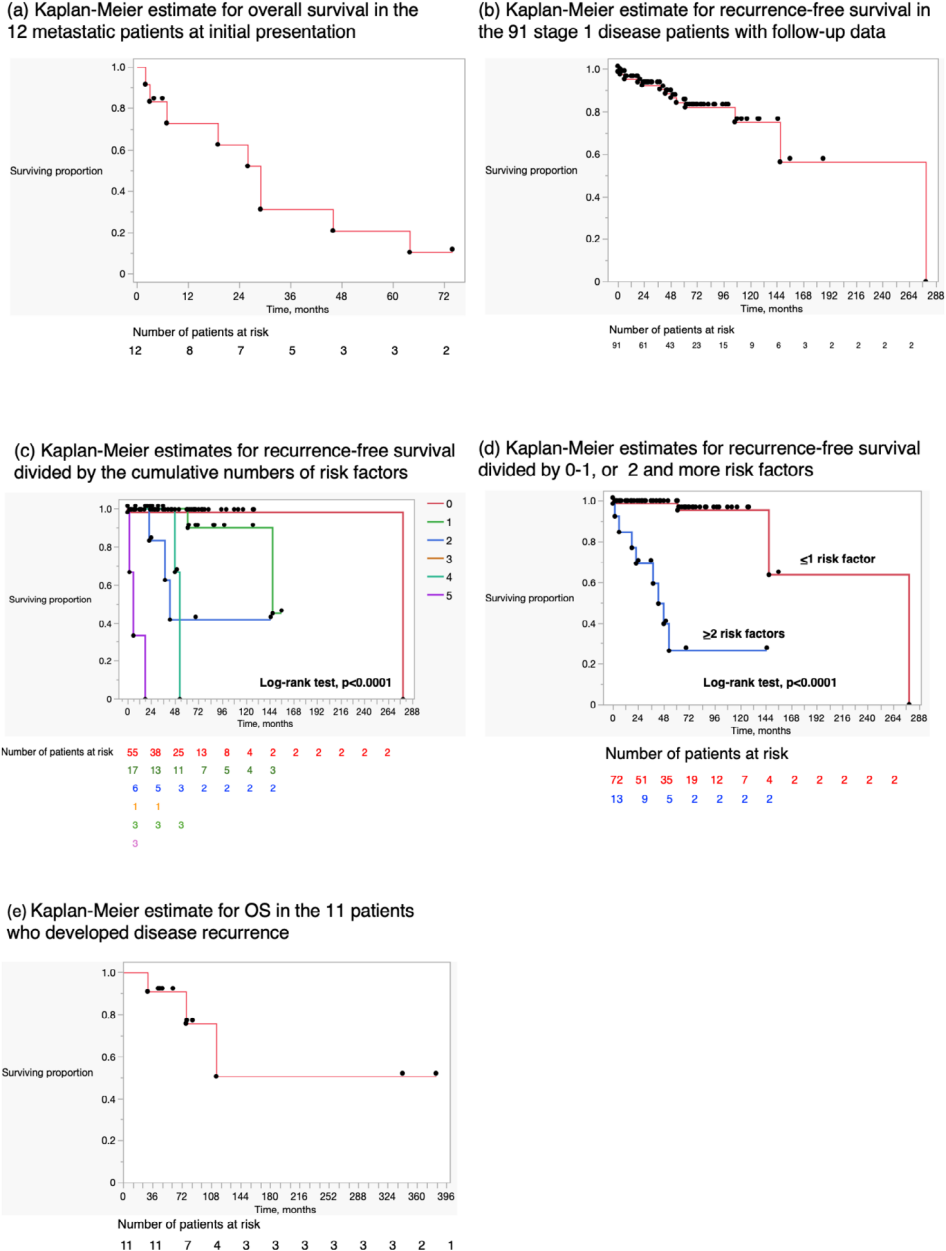
Kaplan–Meier estimates. (a) Kaplan–Meier estimate for overall survival in the 12 metastatic patients at initial presentation. (b) Kaplan–Meier estimate for recurrence‐free survival in the 91 stage 1 disease patients with follow‐up data. (c) Kaplan Meier estimates for recurrence‐free survival divided by the cumulative numbers of the risk factors. (d) Kaplan–Meier estimates for recurrence‐free survival divided by 0–1, or 2 and more risk factors (e) Kaplan–Meier estimate for overall survival in the 11 patients who developed disease recurrence.

Table 3a summarizes the treatments for metastatic disease patients at initial presentation. Most patients (10/12, 83.3%) underwent systemic chemotherapy during the treatment course. The combination including bleomycin, etoposide, and cisplatin was a dominant regimen for first‐line treatment (6/10, 60%), and a taxane‐combination regimen was utilized for second‐line settings. Half of the patients (6/12, 50%) underwent surgery for metastasis, including retroperitoneal/endopelvic LND (*n* = 4), partial lung resection (*n* = 1), and resection of brain metastasis (*n* = 1). Two of them initially underwent RPLND and subsequently received systemic chemotherapy for disease relapse. Three patients underwent radiotherapy, including whole brain irradiation, radiation to abdominal lymph node metastasis, intrapelvic metastasis, and L5 bone metastasis. Figure 3a shows the Kaplan–Meier survival estimate, with a 5‐year OS of 20.8%.

**TABLE 3:** Summary of treatments.

(a) Summary of treatments in the 12 synchronous metastatic patients	
Systemic chemotherapy	
Yes	10
No	2
Chemotherapy regimens	
First line	
Bleomycin, Etoposide, CDDP	6
Etoposide+Ifosphamide+CDDP	1
Etoposide+CDDP	1
Peplomycin Sulfate, Etoposide, CDDP	1
Mitotane	1
Second line	
Docetaxel+Ifosphamide+Nedaplatin	1
Paclitaxel+Ifosohamide+CDDP	1
Paclitaxel+Ifosohamide+Nedaplatin	1
Paclitaxel+CDDP	1
Docetaxel+Ifosphamide+CDDP	1
Third line	
Gemcitabine+Oxaliplatin+Paclitaxel	2
Paclitaxel+CBDCA	1
Fourth line	
Irinotecan+Nedaplatin	1
Paclitaxel+Gemcitabine+CDDP	1
Surgery for metastasis	
Yes	6
No	6
Details of metastasectomy	
Retroperitoneal/endopelvic LND	4
Partial lung resection	2
Resection of brain metastais	1
Radiotherapy for metastasis	
Yes	3
No	9
Details of radiotherapy	
Whole‐brain irradiation	1
To abdominal lymph node metastasis	1
To intrapelvic metastasis	1
To L5 bone metastasis	1

(b) Summary of treatments in the 11 patients with metachronous relapsed disease	
Treatments	No. of patients
Surgery for metastasis	
Yes	9
No	2
Details of metastasectomy	
Retroperitoneal LND	6
LND for other areas	1
Partial lung resection	1
Resection of brain metastasis	1
Resection of small intestine	1
Others	1
Systemic chemotherapy	
Yes	5
No	6
Chemotherapy regimen	
Bleomycin, Etoposide, CDDP	3
Etoposide+Ifosphamide+CDDP	1
Etoposide+CDDP	1
Paclitaxel monotherapy	1
Mitotane	1
Radiotherapy for metastasis	
Yes	3
No	8
Details of radiotherapy	
To lung metastasis	2
To retroperitonel recurrence	1
To brain metastasis	1

Abbreviations: CBDCA, carboplatin; CDDP, cisplatin; LND, lymph node dissection; Nedaplatin, cisplatin analogue developed in Japan.

### 3.4 Long‐Term Outcomes of Stage I Disease

Of the 100 patients who did not have metastasis at the initial presentation, follow‐up data were available in 91. The median follow‐up time was 43 months (range, 0–279). One patient (inflammatory and nested SCT) underwent adjuvant RPLND, revealing cancer‐free lymph nodes, and another patient (Leydig cell tumor) underwent adjuvant chemotherapy comprising 4 cycles of bleomycin, vincristine, and actinomycin D. During the follow‐up, 11 developed disease recurrence (RPLNs = 4, lung = 3, lung+RPLNs = 1, RPLNs+distant LNs = 1, lung+liver+RPLNs = 1, mesentery of small intestine = 1), and three died of other diseases. Recurrence sites were similar to those of testicular germ cell tumors, and the five‐year RFS was 84.4% (Figure 3b). In terms of pathological risk factors on disease relapse, all the abovementioned 6 risk factors were evaluated in the 85 patients. As shown in Table S2, each pathological characteristic had an adverse impact on disease recurrence, although the *p*‐value was marginal with > 5 cm tumor diameter (*p*‐value = 0.0829). Patient age was also a significant risk factor. Figure 3c shows the Kaplan–Meier estimates divided by cumulative numbers of risk factors, showing the recurrence rates rising as the number of pathological risk factors increased. As presented in Figure 3d, patients with > 2 risk factors had shorter RFS than those with < 1 risk factor (5‐year, 26.4 vs. 98.6%, respectively, log‐rank test, *p* < 0.0001). When adjusting for the effect of age on the multivariate model, > 2 risk factors remained significant [hazard ratio = 21.559 (95% CI, 4.256–109.222), *p* = 0.0002], while age did not [hazard ratio = 1.025 (95% CI, 0.993–1.071), *p* = 0.1956].

### 3.5 Treatments and Outcomes in the 11 Patients With Disease Relapse

Table S3 shows patient‐specific treatment courses, and Table 3b summarizes them in the 11 patients with disease relapse. Surgery was most frequently utilized in the recurrent cases (9/11, 81.8%), while systemic chemotherapy was used in 5 patients (5/11, 45.5%). Among them, three (3/11, 27.3%) patients underwent both treatments. In the selected patients (patient Nos. 1, 5, 10), repeated surgeries without systemic chemotherapy or radiotherapy contributed to long‐term disease control. The 5‐ and 10‐year OS rates were 90.9% and 50.5%, respectively (Figure 3e).

## 4 Discussion

In the present retrospective study, through extensive collaborative efforts, we collected the therapeutic outcomes of 112 SCSTs patients; to our knowledge, this is the most comprehensive case series in Japan, revealing clinical features of this rare entity. Regarding clinical manifestations, the most common symptom was scrotal enlargement/testicular induration (55/112, 49.1%), demonstrating a mountain‐shaped distribution with peaks in the 20s and 30s. Furthermore, we observed that incidental detection during infertility assessment or treatment was also frequent (25/112, 22.3%), showing a steep peak in the 30s. We regarded the implementation of central pathological review by the two uropathological experts (100/116, 86.2%) as an additional strength of the present investigation.

Among the 91 stage I patients with available follow‐up data, 11 developed disease recurrence. As shown in Table S2, recurrence sites were similar to those of testicular germ cell tumors, while the timing of recurrence was different. As shown by the Kaplan–Meier curve (Figure 3b), recurrence events occurred continuously up to 5 years, with 3 cases involving recurrences beyond 5 years (at 61, 106, and 147 months, respectively). Our observations suggest that long‐term follow‐up exceeding 5 years should be considered, and physicians should keep in mind that late relapse is not uncommon in stage I SCST patients with adverse pathological characteristics, as described below.

Regarding adverse pathological features associated with malignant clinical behaviors, Kim et al. compared the histological features of 5 malignant and 14 benign Leydig cell tumors, and defined 6 risk factors: tumor greater than 5 cm, necrosis, moderate to severe nuclear atypia, lymphovascular invasion, infiltrative growth, and greater than 3 mitotic figures per 10 high‐power fields [2]. In the current univariate survival analysis, we observed that each histological feature was associated with the disease relapse risk, while the *p*‐value was marginal with a > 5 cm tumor diameter (*p* = 0.0829). As demonstrated in Figure 3d, patients with > 2 risk factors had shorter RFS than those with < 1 risk factor (5‐year rate, 26.4 vs. 98.6%, respectively, log‐rank test, *p* < 0.0001), and this remained significant [hazard ratio = 21.559 (95% CI, 4.256–109.222), *p* = 0.0002] after adjusting for age in the multivariate model. Several investigators reported similar trends. In a systematic review involving 292 stage I SCST patients, Rove et al. demonstrated that, among pathologic risk factors including largest tumor > 5 cm, ≥ 3 mitoses per high‐power field, positive margins, rete testis invasion, lymphovascular invasion, nuclear atypia, and necrosis, 5‐year RFS was 98.1% for those with < 2 risk factors vs. 44.9% for those with ≥ 2 risk factors (*p* < 0.001) [8]. In a retrospective study from Memorial Sloan Kettering of a 15‐year experience, 37 of 48 SCST patients had 0 or 1 pathological risk factor, and they were managed with observation after orchiectomy. None developed disease recurrence, with a median duration of 14.5 months. RPLND was performed in 11 patients, including 6 stage I patients with > 2 risk factors, 2 stage IIA patients at the initial diagnosis, and 3 patients who had > 2 risk factors and developed retroperitoneal recurrence after observation. Regarding the 6 patients undergoing prophylactic RPLND, 4 were disease‐free at a median follow‐up of 6.6 years. Neither of the 2 patients with IIA disease at the initial staging developed relapse, and all 3 patients with delayed RPLND revealed disease relapse and 1 died of disease [7].

In the present study, 12 patients presented with distant disease at the initial staging, demonstrating a poor outcome (5‐year OS of 20.8%). As shown in Table 3, although systemic chemotherapy was most frequently employed, we further confirmed that a conventional chemotherapy regimen for a germ cell tumor had limited efficacy, and metastatic SCSTs were resistant to multimodal treatments. Regarding the prognosis of 11 patients who developed disease recurrence in a metachronous manner, the 5‐ and 10‐year OS rates were 90.9% and 50.5%, respectively. In selected patients (patient Noa.1, 8, and 10), repeated surgeries contributed to long‐term disease control. Survival differences between metachronous and synchronous disease may indicate that low‐volume metastasis detected on routine follow‐up, especially RPLNs, could still be curable by surgical disease elimination. Considering the lack of effective systemic chemotherapy for this rare disease entity, we agree that prophylactic RPLND may be beneficial for stage I SCST patients with > 2 pathological risk factors [7].

Regarding the potential of minimally invasive RPLND for this rare disease entity, Peschel R et al. reported their experience with laparoscopic RPLND within a unilateral template in 6 patients with Leydig cell tumors [9]. The mean operative time was 190 min without open conversion, and pathological analysis of lymph nodes revealed no metastasis. At a mean follow‐up of 12 months, no patient developed disease recurrence. Given the accumulated evidence supporting the feasibility and equivalent oncological outcomes in minimally invasive RPLND (laparoscopic or robotic) [10, 11, 12], we consider that these approaches could be viable options for stage I SCST patients with multiple adverse pathological risks, such as utilizing a full template bilateral nerve‐sparing methodology.

Our study had several limitations, including the small number of metastatic disease patients and retrospective nature, although these represent unavoidable limitations in rare diseases. Since treatment approaches for metastasis or disease recurrence were determined by individual physicians and varied considerably, this heterogeneity may have influenced the survival outcomes. Lack of standardized surveillance protocols for SCSTs, and the absence of reliable tumor markers potentially affect patient compliance, and the generally indolent behavior of SCSTs could contribute to loss to follow‐up, which may have distorted our observations. The relatively small number of recurrence events in our cohort, particularly when stratified into multiple subgroups, may limit the precision of survival estimates and statistical power of group comparisons. Therefore, the findings should be interpreted with caution. Furthermore, owing to the limited sample size of the current cohort and paucity of recurrence events, we did not perform survival analyses stratified by histological subtypes. Upon comparing recurrence‐free survival between stage I Sertoli cell (*n* = 33) and Leydig cell (*n* = 43) tumors, representing the two predominant histological subtypes, no significant difference in survival was observed between the two groups (data not shown). Finally, the present study could not answer whether primary RPLND selectively performed for clinical stage I patients with multiple adverse risk factors could alter the natural course of the disease. Nevertheless, the present study is the most comprehensive case series in Japan, confirming the survival impact of adverse risk factors and significance of surgical disease elimination in selected cases. Our next aim is to construct a digital histopathological database repository to enhance diagnostic accuracy and advance pathological expertise.

## Author Contributions

**Takashige Abe:** conceptualization, formal analysis, writing – original draft, writing – review and editing. **Kosuke Miyai:** investigation. **Toyonori Tsuzuki:** conceptualization, supervision. **Sachiyo Murai:** data curation. **Katsuyoshi Hashine:** investigation. **Shinichi Yamashita:** investigation. **Takashi Kawahara:** investigation. **Naotaka Nishiyama:** investigation. **Hiroshi Kitamura:** supervision. **Hiroshi Kikuchi:** investigation. **Kazutaka Saito:** investigation. **Hatsuki Hibi:** investigation. **Haruka Miyata:** investigation. **Ryuji Matsumoto:** investigation. **Takahiro Osawa:** investigation. **Hiroyuki Nishiyama:** supervision. **Nobuo Shinohara:** supervision.

## Funding

The authors have nothing to report.

## Ethics Statement

Approval of the Research Protocol by an Institutional Reviewer Board: This study was approved by Hokkaido University Hospital Life and Medical Research Ethics Review Committee, No.: 022–0073.

## Consent

Written or oral informed consent was obtained when possible; otherwise, an opt‐out consent method approved by the institutional review board was used, with public disclosure of study information allowing patients to decline participation.

## Conflicts of Interest

Kazutaka Saito is an Editorial Board member of International Journal of Urology and a co‐author of this article. To minimize bias, they were excluded from all editorial decision‐making related to the acceptance of this article for publication.

## Supporting information


**Table S1:** Immunohistochemical panel.

**Table S2:** Univariate analysis of prognostic factors for recurrence‐free survival in the 85 stage 1 patients with data on the 6 adverse pathological characteristics available.

**Table S3:** Patient‐specific treatment courses of the 11 patients with disease relapse.

## References

[1] J. P. Dilworth , G. M. Farrow , and J. E. Oesterling , “Non‐Germ Cell Tumors of Testis,” Urology 37 (1991): 399–417. 2024387 10.1016/0090-4295(91)80100-l

[2] I. Kim , R. H. Young , and R. E. Scully , “Leydig Cell Tumors of the Testis. A Clinicopathological Analysis of 40 Cases and Review of the Literature,” American Journal of Surgical Pathology 9 (1985): 177–192. 3993830 10.1097/00000478-198503000-00002

[3] J. S. Banerji , K. Odem‐Davis , E. M. Wolff , C. R. Nichols , and C. R. Porter , “Patterns of Care and Survival Outcomes for Malignant Sex Cord Stromal Testicular Cancer: Results From the National Cancer Data Base,” Journal of Urology 196 (2016): 1117–1122. 27036305 10.1016/j.juro.2016.03.143

[4] J. Grogg , K. Schneider , P. K. Bode , et al., “Sertoli Cell Tumors of the Testes: Systematic Literature Review and Meta‐Analysis of Outcomes in 435 Patients,” Oncologist 25 (2020): 585–590. 32043680 10.1634/theoncologist.2019-0692 PMC7356704

[5] C. D. Fankhauser , J. B. Grogg , S. Hayoz , et al., “Risk Factors and Treatment Outcomes of 1,375 Patients With Testicular Leydig Cell Tumors: Analysis of Published Case Series Data,” Journal of Urology 203 (2020): 949–956. 31845841 10.1097/JU.0000000000000705

[6] J. B. Grogg , K. Schneider , P. K. Bode , et al., “Risk Factors and Treatment Outcomes of 239 Patients With Testicular Granulosa Cell Tumors: A Systematic Review of Published Case Series Data,” Journal of Cancer Research and Clinical Oncology 146 (2020): 2829–2841. 32719989 10.1007/s00432-020-03326-3 PMC7519920

[7] J. L. Silberstein , W. M. Bazzi , E. Vertosick , et al., “Clinical Outcomes of Local and Metastatic Testicular Sex Cord‐Stromal Tumors,” Journal of Urology 192 (2014): 415–419. 24518791 10.1016/j.juro.2014.01.104 PMC6701173

[8] K. O. Rove , P. D. Maroni , C. R. Cost , et al., “Pathologic Risk Factors for Metastatic Disease in Postpubertal Patients With Clinical Stage I Testicular Stromal Tumors,” Urology 97 (2016): 138–144. 27538802 10.1016/j.urology.2016.06.066

[9] R. Peschel , M. T. Gettman , H. Steiner , R. Neururer , and G. Bartsch , “Management of Adult Leydig‐Cell Testicular Tumors: Assessing the Role of Laparoscopic Retroperitoneal Lymph Node Dissection,” Journal of Endourology 17 (2003): 777–780. 14642042 10.1089/089277903770802362

[10] P. Pongratanakul , M. Vermeulen‐Spohn , C. Woltjen , et al., “Matched‐Pair Analysis of Peri‐Operative and Oncological Outcomes of Robot‐Assisted vs Open Retroperitoneal Lymph Node Dissection,” BJU International 136 (2025): 150–158. 40260829 10.1111/bju.16747 PMC12134411

[11] J. Lin , Z. Hu , S. Huang , et al., “Comparison of Laparoscopic, Robotic, and Open Retroperitoneal Lymph Node Dissection for Non‐Seminomatous Germ Cell Tumor: A Single‐Center Retrospective Cohort Study,” World Journal of Urology 41 (2023): 1877–1883. 37332060 10.1007/s00345-023-04459-z PMC10352171

[12] S. Stepanian , M. Patel , and J. Porter , “Robot‐Assisted Laparoscopic Retroperitoneal Lymph Node Dissection for Testicular Cancer: Evolution of the Technique,” European Urology 70 (2016): 661–667. 27068395 10.1016/j.eururo.2016.03.031

