# Supplementary material for: Current Treatment Patterns and Outcomes of Sex Cord Stromal Tumor Patients in Japan
Source: Int J Urol. 2026 Mar 10;33(3):e70399. doi: 10.1111/iju.70399 (PMC12976466; doi:10.1111/iju.70399)
Supplement: Supplementary file 1 — Table S1: Immunohistochemical panel. [file IJU-33-0-s002.docx]

Supplementary Table 1. Immunohistochemical panel

| Antibody | Clonality (clone) | Dilution | Source |
| --- | --- | --- | --- |
| Inhibin | Mouse monoclonal (R1) | 1:40 | Dako |
| Steroid factor-1 | Mouse monoclonal (N1665) | 1:30 | Thermo Fisher Scientific |
| Melan A | Mouse monoclonal (A103) | 1:50 | Novocastra |
| Calretinin | Rabbit monoclonal (SP13) | 1:300 | Cell Marque |
| β-catenin | Rabbit monoclonal (E247) | 1:200 | Origene Technologies |
| CD30 | Mouse monoclonal (Ber-H2) | 1:60 | Dako |
| Synaptophysin | Mouse monoclonal (27G12) | 1:300 | Novocastra |
| Chromogranin A | Mouse monoclonal (DAK-A3) | 1:200 | Dako |
| SALL4 | Mouse monoclonal (6E3) | 1:100 | Cell Marque |
