# Supplementary material for: Current Treatment Patterns and Outcomes of Sex Cord Stromal Tumor Patients in Japan
Source: Int J Urol. 2026 Mar 10;33(3):e70399. doi: 10.1111/iju.70399 (PMC12976466; doi:10.1111/iju.70399)
Supplement: Supplementary file 2 — Table S2: Univariate analysis of prognostic factors for recurrence‐free survival in the 85 stage 1 patients with data on the 6 adverse pathological characteristics available. [file IJU-33-0-s003.docx]

**Supplementary Table 2. Univariate analysis of prognostic factors for recurrence-free survival in the 85 stage 1 patients with data on the 6 adverse pathological characteristics available**

| **Variables** | **Univariate analysis, hazard ratio (95% CI)** | **p-value** |
| --- | --- | --- |
| **Age, year** |  |  |
| Continuous | 1.044 (1.010-1.084) | 0.016 |
| **Tumor size** |  |  |
| <5 cm | 1 |  |
| >5 cm | 3.068 (0.864-10.892) | 0.0829 |
| **Lymphovascular invasion** | |  |
| No | 1 |  |
| Yes | 12.288 (3.518-42.921) | <0.0001 |
| **>3 mitotic features per 10 high-power fields** |  |  |
| No | 1 |  |
| Yes | 7.287 (2.185-24.305) | 0.0012 |
| **Nuclear atypia** |  |  |
| No | 1 |  |
| Yes | 6.778 (1.895-24.238) | 0.0032 |
| **Necrosis** |  |  |
| No | 1 |  |
| Yes | 11.148 (3.157-39.364) | 0.0002 |
| **Infiltrating growth** |  |  |
| No | 1 |  |
| Yes | 20.662 (5.826-73.278) | <0.0001 |
