# Supplementary material for: Current Treatment Patterns and Outcomes of Sex Cord Stromal Tumor Patients in Japan
Source: Int J Urol. 2026 Mar 10;33(3):e70399. doi: 10.1111/iju.70399 (PMC12976466; doi:10.1111/iju.70399)
Supplement: Supplementary file 3 — Table S3: Patient‐specific treatment courses of the 11 patients with disease relapse. [file IJU-33-0-s001.docx]

**Supplementary Table 3. Patient-specific treatment courses of the 11 patients with disease relapse**

| **Patient** | **Pathology** | **No. of adverse pathologic features** | **Relapse site** | **Recurrence-free duration (months)** | **Surgery for metastasis** | **Systemic chemotherapy** | **Radiotherapy** | **Outcome** | **Total follow-up duration (months)** |
| --- | --- | --- | --- | --- | --- | --- | --- | --- | --- |
| 1 | Leydig cell tumor | at least 1 | RPLNs | 106 | RPLND x2 | - | - | NED | 383 |
| 2 | Leydig cell tumor | 4 | RPLNs, and lung | 48 | RPLND and nephrectomy | - | To retroperitonal relapse, and lung metastasis | AWC | 78 |
| 3 | Leydig cell tumor | 2 | RPLNs | 43 | RPLND | - | - | AWC | 48 |
| 4 | Leydig cell tumor | 5 | RPLNs, and paraesophageal lymph nodes | 18 | RPLND, and resection of paraesophageal lymph nodes | Bleomycin+etoposide+ CDDP, mitotane | - | AWC | 43 |
| 5 | Sertoli cell tumor | 1 | RPLNs | 147 | RPLND x3 | - | - | NED | 342 |
| 6 | Sertoli cell tumor | 5 | RPLNs | 6 | RPLND | Etoposide + CDDP, paclitaxel monotherapy | - | DOD | 77 |
| 7 | Sertoli cell tumor | 2 | Lung | 22 | - | Bleomycin+etoposide+ CDDP | - | AWC | 45 |
| 8 | Large cell calcifying Sertoli cell tumor | 1 | Lung | 61 | Lobectomy | - | - | NED | 85 |
| 9 | Sertoli-Leydig cell tumor | at least 2 | Lung | 6 | Resection of brain metastasis | Bleomycin+etoposide+ CDDP | Total-brain irradiation | DOD | 30 |
| 10 | Inflammatory and nested sex cord tumor | 4 | Mesentery of small intestine | 53 | Resection of mesentery disese x2, and resection of subdiaphragmatic recurrrence | - | - | AWC | 114 |
| 11 | Unclassified sex cord stromal tumor | 5 | Lung, liver, and RPLNs | 2 | - | Etoposide+ifosphamide+CDDP | To lung metastais | NED | 61 |
